# Hybrid Mesoporous Carbon/Copper Ferrite Electrode for Asymmetric Supercapacitors

**DOI:** 10.3390/nano13162365

**Published:** 2023-08-18

**Authors:** Khang Huynh, Bharathkiran Maddipudi, Rajesh Shende

**Affiliations:** Karen M. Swindler Department of Chemical and Biological Engineering, South Dakota School of Mines & Technology, Rapid City, SD 57701, USA; khang.huynh@mines.sdsmt.edu (K.H.); bharathkiran.maddipudi@mines.sdsmt.edu (B.M.)

**Keywords:** Cu-ferrite, mesoporous carbon, asymmetric supercapacitor, CV, GCD, electrochemical impedance spectroscopy

## Abstract

Asymmetric supercapacitors (ASCs) with two dissimilar electrodes are known to exhibit relatively moderate energy and power densities. If electrodes derived from earth-abundant materials or renewable resources such as lignocellulosic biomass (LCB) are used for fabrication, energy storage systems are expected to become less expensive and more sustainable. Hybrid electrode materials have advantages such as higher surface area, better chemical stability, and superior energy density. This study reports on the synthesis of a novel hybrid electrode material containing porous carbon (POC) and copper ferrite, which is designated as POC@Cu-ferrite, and its electrochemical performance in ASC configuration. Corn stover derived hydrochar is utilized for the sol–gel synthesis of POC@Cu-ferrite hybrid material using earth-abundant Cu and Fe-based precursors. This material is characterized using X-ray diffraction (XRD), Raman spectroscopy, Brunauer–Emmett–Teller (BET) surface area analyzer, and scanning and transmission electron microscopy (SEM/TEM). As-synthesized Cu-ferrite is found to contain 89.2% CuFe_2_O_4_ and 10.8% Fe_2_O_3_, whereas other phases such as Fe_3_O_4_, CuFeO_2_, and CuO are observed for the POC@Cu-ferrite. BET-specific surface area (SSA) and pore volume of POC@Cu-ferrite are observed as 1068 m^2^/g and 0.72 cm^3^/g, respectively. POC@Cu-ferrite hybrid electrode is used with POC opposite electrode to fabricate ASC, which is tested using Gamry G-300 potentiostat/galvanostat/ZRA to obtain cyclic voltammetry (CV) profiles and galvanostatic charge–discharge (GCD) plots. ASC is also prepared using Cu-ferrite and POC materials and its specific capacitance and stability are compared with ASCs prepared with POC@Cu-ferrite and POC or graphene nanoplatelets (GNPs) electrodes. POC@Cu-ferrite hybrid electrode is found to be superior with a 2-fold higher capacitance and significant electrochemical stability over 100 GCD cycles as compared to the Cu-ferrite electrode.

## 1. Introduction

Energy storage materials for devices such as Zn-ion batteries [1,2], Li-Se batteries [3], Li-ion batteries [4], ammonium-ion batteries [5], and supercapacitors [6] have been extensively investigated in recent years. Asymmetric supercapacitor (ASC) (a special kind with two dissimilar electrodes) has attracted a lot of attention from researchers due to its superior power and energy densities as compared to traditional electric double-layer capacitors (EDLCs), and faster charging/discharging rate as compared to batteries [7]. Typically, ASC is fabricated with a negative electrode with capacitive behavior and a positive electrode with a Faradaic response [8].

Recently, spinel ferrite nanomaterials (SFNs) such as LiFe_2_O_4_ [9], CoFe_2_O_4_ [10,11,12], NiFe_2_O_4_ [13,14,15], and CuFe_2_O_4_ have been reported as better electrode materials for ASCs because of their superior electrochemical performance. CuFe_2_O_4_ (Cu-ferrite) is considered as a better material for energy storage application because of its unique valence shell electronic configuration (3d^10^4s^1^), higher theoretical capacity (895 mAh/g), and relatively low cost due to the abundance of Cu and Fe [16,17,18,19]. Its performance can be further enhanced by combining it with carbonaceous material leading to hybrid electrode configurations. Zhang et al. [20] incorporated graphene nanosheet into CuFe_2_O_4_ and reported enhanced electrochemical capacitance of 576.6 F/g at 1 A/g as compared to CuFe_2_O_4_. Moreover, CuFe_2_O_4_/graphene exhibited a higher electrochemical stability of 85% over 300 charging–discharging cycles as compared to CuFe_2_O_4_, which showed stability of only 58% [20]. Guo et al. [21] used a nonionic surfactant (Tween-80) to modify the structure of CuFe_2_O_4_. This modified CuFe_2_O_4_ electrode material showed a maximum specific capacitance value of 437.3 F/g at 0.004 V/s scan rate as compared to 73.6 F/g observed for the CuFe_2_O_4_ electrode. Tween-80-modified CuFe_2_O_4_ also showed smaller bulk resistance and higher stability in comparison to CuFe_2_O_4_ nanoparticles [21]. Thus, the use of carbonaceous materials with ferrite and surfactant appear to improve the electrochemical performance.

Although numerous methods have been reported to modify the CuFe_2_O_4_ materials for better electrochemical performance [22,23,24,25], studies on the use of POC derived from renewable LCB for the preparation of hybrid electrodes are not available in the literature. POC derived from agriculture waste, animal waste, and municipal waste have been investigated as possible alternatives to industrially carbonaceous materials such as graphene, carbon nanotubes (CNTs) [26,27], and reduced graphene oxide (rGO) [28,29]. Some of these carbonaceous materials, for instance, hydrochar or biochar can be obtained from LCB via thermochemical processes such as pyrolysis, hydrothermal carbonization/liquefaction (HTC/HTL). Hydrochar/biochar can be subjected to chemical activation and thermal treatment to obtain a POC with superior pore volume, porosity, and specific surface area (SSA) [30,31,32]. This highly porous POC material can be combined with SFNs to prepare hybrid nanomaterials that can exhibit superior electrochemical performance [33,34,35].

In this investigation, a novel hybrid POC@Cu-ferrite electrode material is prepared via the sol–gel synthesis approach using POC, and Cu and Fe precursors. The hybrid electrode is used in ASC configuration and its electrochemical performance is measured and compared with Cu-ferrite electrodes.

## 2. Materials and Methods

### 2.1. Materials

Copper (II) chloride (CuCl_2_.2H_2_O, 99%, CAS number 10125-13-0, Alfa Aesar, Haverhill, MA, USA) and iron (II) chloride (FeCl_2_.4H_2_O, 98%, CAS number 13478-10-9, Alfa Aesar, Haverhill, MA, USA) were used for the synthesis of Cu-ferrite. Ethanol (200 Proof, CAS number 64-17-5) was purchased from Pharmco Aaper Products, Brookfield, CT, USA. Pluronic P123 surfactant (CAS number 9003-11-6) and propylene oxide (CH_3_CHCH_2_O, 99%, CAS number 75-56-9) were purchased from Sigma Aldrich, St. Louis, MO, USA. Idaho National Laboratory (INL) supplied the preprocessed corn stover feedstock (avg. size of 1.12 mm). For the activation of hydrochar, analytical-grade KOH (90%, CAS number 1310-58-3, Sigma Aldrich, St. Louis, MO, USA) and hydrochloric acid (HCl, 35.5%, CAS number 7647-01-0, Fisher-Scientific, Hampton, NH, USA) were used. Polytetrafluoroethylene (PTFE 60 wt.% dispersion in water, CAS number 9002-84-0, Sigma Aldrich, St. Louis, MO, USA), Super P conductive carbon black (CAS number 1333-86-4, MSE Supplies, Tucson, AZ, USA), and nickel foam substrates (1.6 mm thick, 99.9% purity, MTI Corporation, Richmond, CA, USA) were utilized for the preparation of working electrodes.

### 2.2. Synthesis of POC

To carry out HTC/HTL, preprocessed corn stover (15 g) was loaded into a 300 mL PARR SS316 reactor with 150 mL de-ionized (DI) water and heated to 250 °C for 1 h. After HTC/HTL processing, the solid residue (hydrochar) was filtered and extracted with acetone to separate and recover bio-oil from the hydrochar. The hydrochar was then dried in oven at 60 °C for 12 h and chemically activated with KOH using hydrochar to KOH weight ratio of 1:5. Chemically activated hydrochar was thermally treated at 800 °C for 1 h under a constant flow of N_2_ (UHP grade). Activated hydrochar was treated with 0.1 M HCl to separate residual impurities. Finally, it was dried at 60 °C in a conventional oven. Additional details about POC preparation can be found elsewhere [28,30,31,32,36,37,38,39,40].

### 2.3. Synthesis of Hybrid POC@Cu-Ferrite Nanomaterial

**Figure 1:**
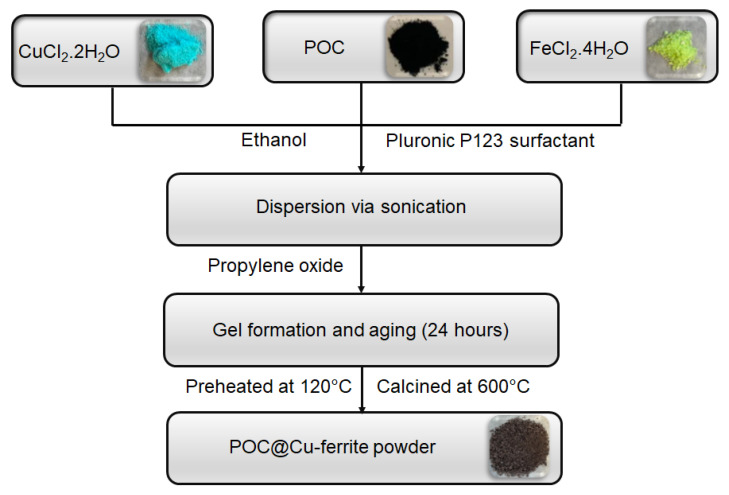
Sol–gel synthesis of hybrid POC@Cu-ferrite nanomaterial.

Briefly, 4.5 g of copper (II) chloride, 10.5 g of iron (II) chloride, 0.05 g of Pluronic P123 surfactant, and 0.5 g of POC were mixed in 30 mL of ethanol and sonicated for 2 h to obtain a dispersion. Gel formation was accomplished with the addition of 30 mL propylene oxide to the dispersion. The gel was aged for 24 h and calcined at 600 °C (ramp rate of 20 °C/min) for 8 h. This material is designated as POC@Cu-ferrite. Synthesis steps for the preparation of POC@Cu-ferrite are depicted in Figure 1. Cu-ferrite was prepared with the similar sol–gel chemistry approach without using POC.

### 2.4. Characterization of POC, Cu-Ferrite, and POC@Cu-Ferrite Nanomaterials

BET analyzer (Micromeritic Gemini II-2375, Norcross, GA, USA) was used to obtain the adsorption/desorption profiles and to determine the pore size, SSA, and pore volume. To prepare samples for BET analysis, 0.15 g of material was degassed under a constant flow of UHP grade N_2_ gas at 200 °C for 2 h in a BET tube. The tube was cooled down to room temperature and loaded into a Dewar flask filled with liquid nitrogen. Adsorption-desorption isotherms were generated in the range of 0 to 1 relative pressure (P/P_0_) to determine SSA, pore area and pore volume.

Cu-ferrite and POC@Cu-ferrite materials were characterized using powdered X-ray diffractometer (operating conditions: 45 kV, 40 mA; Empyrean Series 3, Malvern, UK) provided with a CoKα radiation (wavelength = 1.78899 Å). To accomplish this, 0.15 g of a powdered material was finely crushed with a mortar and pestle and loaded onto the sample holder. Powdered X-ray diffraction analysis was carried out in the 2θ range of 5° ≤ 2θ ≤ 80° with 0.09° per second scan rate. Crystalline phase of a material was determined via the MDI/JADE version 6.5 software. Crystallite size was determined using Scherrer’s equation (Equation (1)),

(1) $$\tau =\frac{K\lambda }{\beta cos\theta }$$

where *K* is the dimensionless shape factor, *τ* is the mean crystalline size, *λ* is the X-ray wavelength, *β* is the wavelength of full-width at half maximum (FWHM), and *θ* is the Bragg angle.

ffTA FORAM X3 Raman Spectroscopy instrument (532 nm laser wavelength, 200 s scan time, Worcestershire, UK) was used to analyze the Cu-ferrite and POC@Cu-ferrite nanomaterials. The analysis was performed to understand the D and G bands in POC@Cu-ferrite and to determine the crystalline planer size.

Morphology of the materials was analyzed via scanning electron microscopy with energy dispersive X-ray spectroscopy (SEM/EDX) using the ThermoFisher Scientific Axia ChemiSEM instrument (3 nm @ 30 kV electron beam resolution, San Diego, CA, USA).

TEM with selected area electron diffraction (SAED) analyses was performed using JEOL JEM-2100 LaB6 instrument (operating conditions: 200 kV accelerating voltage, 109 μA beam current, Peabody, MA, USA).

### 2.5. Fabrication of ASC and Electrochemical Evaluation

The working electrode was prepared using a mixture of Cu-ferrite or POC@Cu-ferrite, PTFE, and Super P with a mass ratio of 8:1:1, respectively. This powder mixture was turned into a viscous slurry using few drops of 18.2 MΩ water and coated onto a nickel foam substrate. The coated substrate was dried in a vacuum oven at 60 °C for 12 h and a chemical resistance epoxy was applied. The working electrode along with a Ag/AgCl reference electrode and a Pt wire counter electrode were used in a typical 3-cell setup to determine the electrochemical performance. Additional details can be found elsewhere [31,40].

ASC was fabricated using two identical copper plates (dimensions: 0.5 in × 0.5 in × 0.025 in thickness). These plates were coated with 0.1 mg electrode material previously mixed with polyurethane (MINWAX). Nylon 6,6 separator was dipped in 8M potassium hydroxide electrolyte and placed between the copper plates. The fabricated assembly was secured with a regular polyethylene film and duct tape to avoid contamination. It was charged with 2–4 volts and 0.003–0.005 amperes using a DC power supply. Lower power was used to avoid any damage to the electrode. Gamry’s G-300 potentiostat/galvanostat/ZRA system was used to characterize the ASCs. Electrochemical performance was measured using CV and GCD profiles. CV measurement was performed by varying the scan rates. GCD measurement was performed with varying the current density. Specific capacitance was calculated using Equation (2),

(2) $$C_{s}=\frac{2\cdot \int_{V_{i}}^{V_{f}}IdV}{m\cdot \left(\frac{dV}{dt}\right)\cdot \left(V_{f}-V_{i}\right)}$$

in which *Cs* is specific capacitance (F/g), *V_i_* and *V_f_* are initial and final voltage sweep values, respectively, *dV*/*dt* is the voltage scan rate (V/s), *m* is the mass of the electrodes (grams), and *I* is the current (amps). Additional details about CV measurements and GCD plots can be found elsewhere [28,36,41,42,43]. For the identification of as-fabricated ASCs with different electrode materials, refer to Table 1.

## 3. Results and Discussion

### 3.1. XRD Spectra of Ferrite Materials

**Figure 2:**
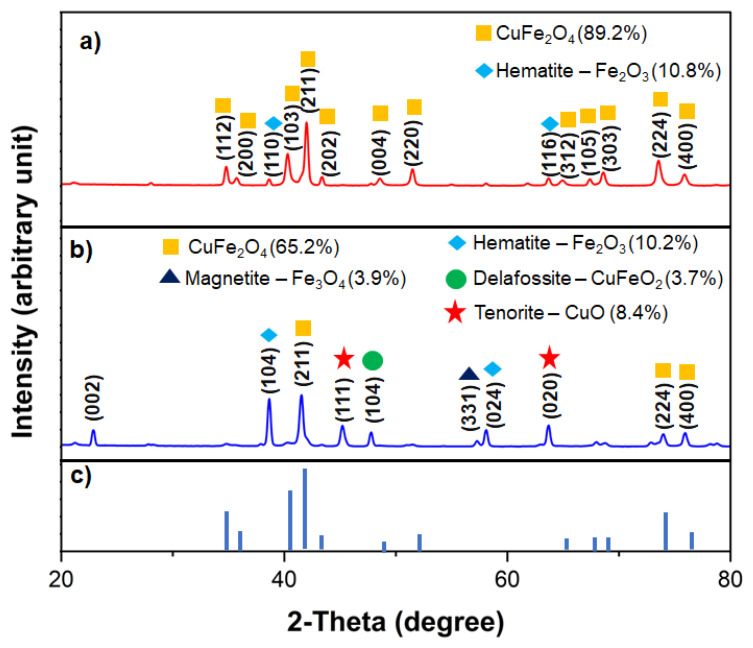
XRD patterns for (**a**) Cu-ferrite, (**b**) POC@Cu-ferrite samples, and (**c**) ICDD standard reflections for CuFe_2_O_4_.

XRD spectra of Cu-ferrite and POC@Cu-ferrite nanomaterials are shown in Figure 2. The spectra for the Cu-ferrite showed the 2θ reflections of 35.7°, 40.3°, 41.9°, 68.4°, 73.4°, and 75.7° with corresponding (*h l k*) values of (1 1 2), (1 0 3), (2 1 1), (2 2 0), (3 0 3), (2 2 4), and (4 0 0), respectively. These 2θ reflections correspond to the tetragonal CuFe_2_O_4_ as per the International Centre for Diffraction Data (ICDD). Additional peaks of 41.5° and 63.6° can be assigned to Fe_2_O_3_ (hematite) as per the ICDD patterns. Overall, the sample consists of 89.2% and 10.8% crystalline phases of Cu-ferrite and hematite, respectively. For the POC@Cu-ferrite material, an additional peak around 23° (0 0 2) corresponds to the graphitic carbon in POC, which is believed to influence the ASC performance [20,44]. The POC@Cu-ferrite material was found to contain 65.2% of Cu-ferrite, 8.6% of carbon, 10.2% of hematite (Fe_2_O_3_), 8.4% of tenorite (CuO), 3.9% of magnetite (Fe_3_O_4_) and 3.7% of delafossite (CuFeO_2_) based on the 2θ reflections reported in ICDD. Using Scherrer’s equation, the crystallite size of Cu-ferrite and POC@Cu-ferrite nanomaterials were calculated as 22 and 15 nm, respectively.

### 3.2. SSA, Pore Volume, and Pore Size of Electrode Materials per BET Analysis

**Figure 3:**
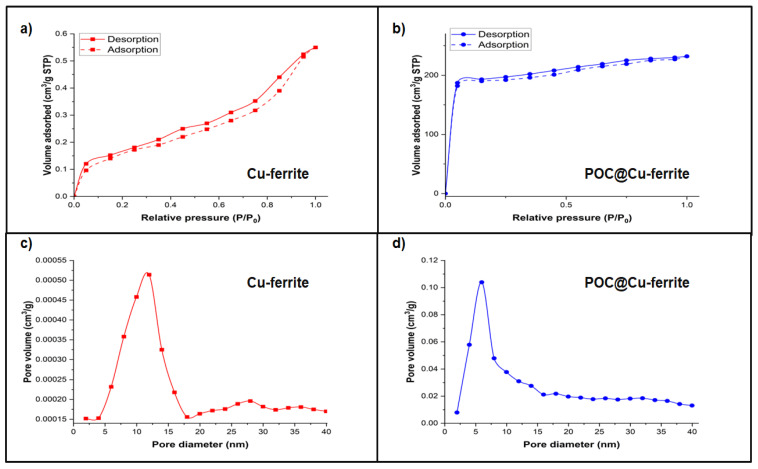
N_2_ adsorption/desorption isotherms for (**a**) Cu-ferrite and (**b**) POC@Cu-ferrite; and pore volume distribution for (**c**) Cu-ferrite and (**d**) POC@Cu-ferrite.

Figure 3 shows the N_2_ adsorption/desorption isotherms for the electrode materials. Cu-ferrite shows a typical type II isotherm (Figure 3a) that corresponds to the non-porous or microporous solids. The POC@Cu-ferrite sample displays a type IV isotherm (Figure 3b). A hysteresis loop is observed due to capillary condensation in the mesoporous structure. Specific surface area (SSA) of the Cu-ferrite and POC@Cu-ferrite from the BET isotherms was determined as 4.7 m^2^/g and 1068 m^2^/g, respectively. SSA of the POC material alone was 1607 m^2^/g; additional details can be found elsewhere [40]. The pore volume was determined as 0.00068 cm^3^/g, 0.86 cm^3^/g, and 0.72 cm^3^/g for the Cu-ferrite, POC, and POC@Cu-ferrite materials, respectively.

Figure 3c,d show the pore volume distribution. It is evident that the POC@Cu-ferrite sample has a pore volume distribution in the range of 2.5–40 nm, which confirms the mesoporous structure. The average pore size of POC@Cu-ferrite was observed as 4.5 nm. It is to be noted that the average pore size for POC was 3.8 nm, which was reported elsewhere [40]. The mesoporous structure of POC@Cu-ferrite can facilitate the redox reactions in ASC and improve the ion diffusivity across the electrode/electrolyte interface [45].

### 3.3. Raman Spectroscopy

**Figure 4:**
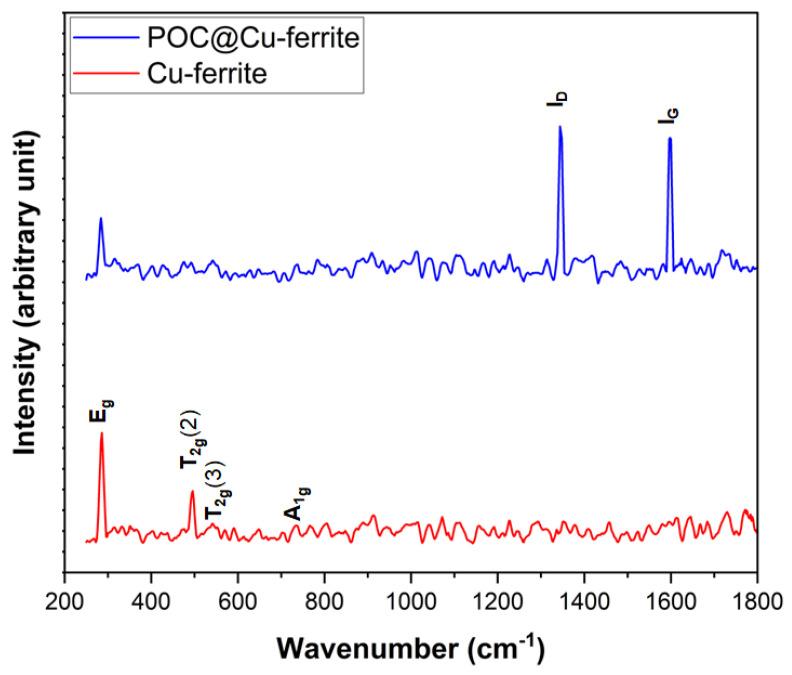
Raman spectra for Cu-ferrite and POC@Cu-ferrite nanomaterials.

Figure 4 shows the Raman spectra for both Cu-ferrite and POC@Cu-ferrite materials. Four different Raman active modes of E_g_, T_2g_(2), T_2g_(3), and A_1g_ at 275, 494, 545, and 706 cm^−1^, respectively, can be observed for Cu-ferrite. These modes are, characteristics of the tetragonal structure, which is formed by the distortion of oxygen and metal ions in Cu-ferrite at the tetrahedral and octahedral sites [46,47,48]. Most notably, the peak at 494 cm^−1^ is assigned to characteristics oxygen vibration in the octahedral site of Cu-ferrite [49]. The Raman spectrum for the POC@Cu-ferrite sample shows two distinct peaks at 1350 and 1600 cm^−1^, which corresponds to the D (defected and disorganized carbon structure) and G-bands (C-C graphitic stretching), respectively [50,51]. Defects in carbon structure can improve the electrochemical performance due to faster electrolyte penetration and better ion storage ability [52]. Moreover, the D-band to G-band intensity ratio (I_D_/I_G_) shows a value of 1.09 for the POC@Cu-ferrite material with the planer size [53] of 4.0 nm.

### 3.4. SEM and TEM Analysis

**Figure 5:**
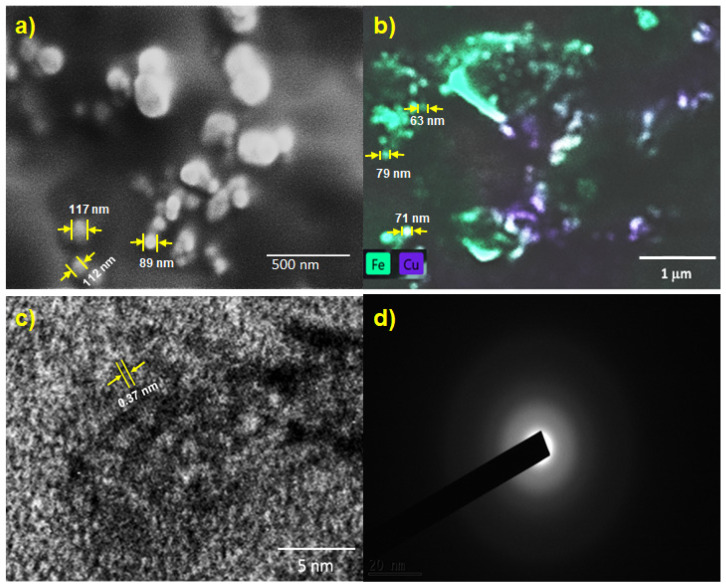
(**a**) SEM image of Cu-ferrite, (**b**) EDX mapping of POC@Cu-ferrite, (**c**) TEM image of POC, and (**d**) SAED pattern of POC.

SEM images of Cu-ferrite and POC@Cu-ferrite materials are shown in Figure 5a,b, respectively. A spherical morphology can be observed for Cu-ferrite. The avg. particle size was observed as 115 nm, which was measured using the ImageJ software (Version 1.53t). SEM image and EDX mapping of POC@Cu-ferrite also indicate spherical nanoparticle morphology and distribution of Cu and Fe on the POC surface with an average particle size of 74 nm. The smaller size of Cu-ferrite nanoparticles in POC@Cu-ferrite suggest that the POC surface provided some resistance for nucleation and growth during gel formation and subsequent dehydration/decarboxylation while drying/calcination. TEM image (Figure 5c) of POC indicates mesoporous morphology with lattice *d*-spacing of about 0.37 nm, which is consistent with the carbon derived from biomass reed flowers [54]. The SAED pattern (Figure 5d) shows no bright spots, which is a characteristic of amorphous carbon.

### 3.5. Electrochemical Performance of ASCs

**Figure 6:**
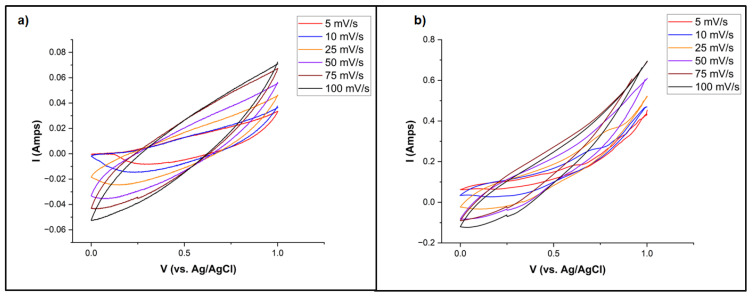
CV profiles for (**a**) Cu-ferrite and (**b**) POC@Cu-ferrite at different scan rates.

Electrochemical testing was carried out with scan rates of 5–100 mV/s between 0 and 1 V voltage window with the purpose of identifying optimum measuring conditions to accurately determine the specific capacitance. Figure 6a,b show CV profiles for the Cu-ferrite and POC@Cu-ferrite materials. No redox peaks can be observed on the CV profiles, which indicate irreversible reactions. At different scan rates, the nature of CV profiles is similar, suggesting stable electrochemical performance. The cyclic voltammograms for POC@Cu-ferrite show a higher area under the curve as compared to Cu-ferrite, suggesting better electrochemical performance. The calculated specific capacitance values for Cu-ferrite and POC@Cu-ferrite were 23–85 F/g and 139–178 F/g, respectively, at different scan rates. As the cell operating voltage used in this study is <1 V, no appreciable gas evolution and damage to ASC are expected. However, evolution of gases such as CO, CO_2_, H_2,_ etc., during charging/discharging process and damage to cell assembly, especially at higher voltages, have been reported in the literature [55].

**Figure 7:**
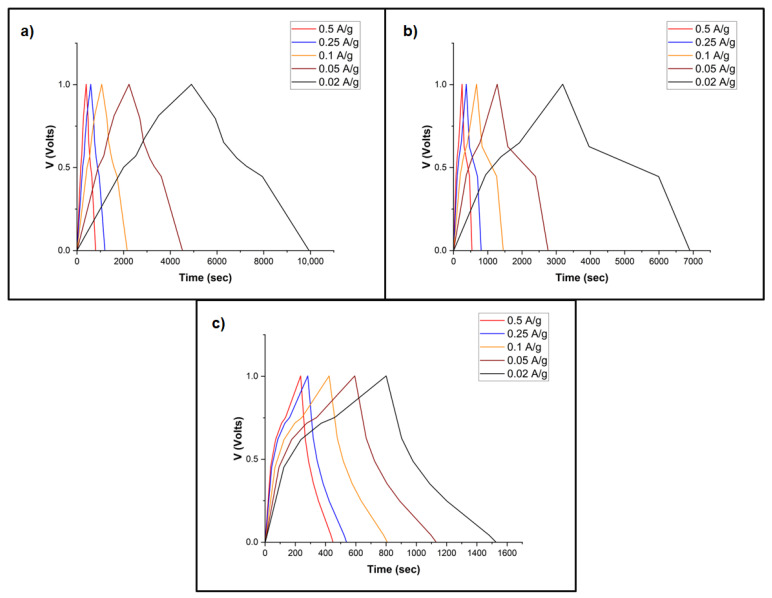
GCD profiles for (**a**) ASC-1, (**b**) ASC-2, and (**c**) ASC-3.

Figure 7 shows the GCD profiles for the ASCs at various current densities of 0.02–0.5 A/g. The GCD curves show symmetrical features in both charging and discharging regions, in which an IR drop at the transition region between charging and discharging occurs due to the equivalent series resistance (ESR) between the interface of the electrode and electrolyte [56]. The GCD profiles do not show typical sawtooth shape similar to EDLCs. It can be observed that it takes 800, 615, and 430 s to complete one GCD cycle at 0.5 A/g for ASC-1, ASC-2, and ASC-3, respectively. Using the current density of 0.02 A/g, the time needed to complete one charging/discharging cycle for ASC-1, ASC-2, and ASC-3 was 9900, 6900, and 1530 s, respectively. As ASC-1 takes longest time to complete one charging/discharging cycle, superior electrochemical performance and higher specific capacitance of this ASC is expected as compared to ASC-2 and ASC-3 [57].

**Figure 8:**
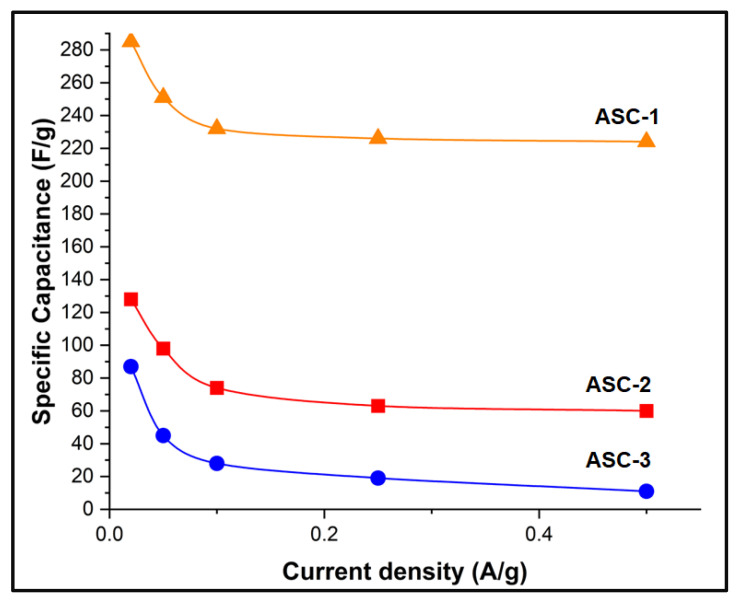
Specific capacitance of the ASCs measured as a function of current density.

**Figure 9:**
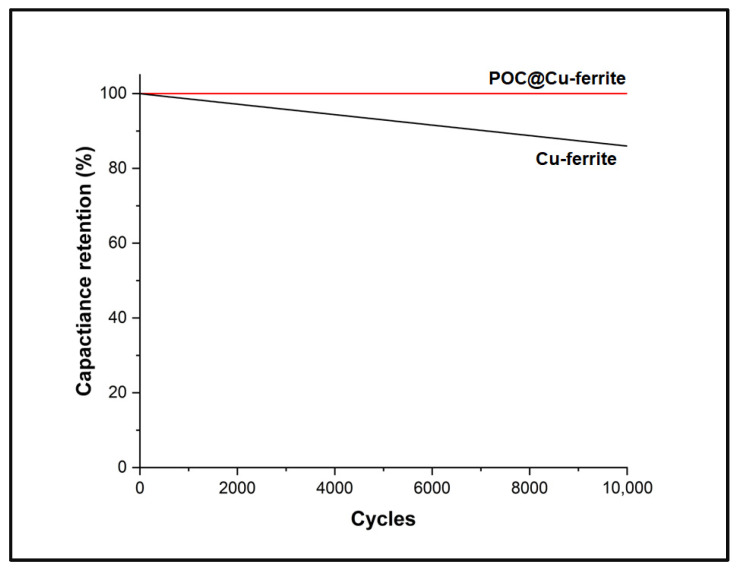
Cycling stability of the working electrodes at 0.5 A/g for 10,000 cycles.

Current densities were varied during electrochemical testing of ASCs, and the specific capacitance values are presented in Figure 8. The specific capacitance is found to be retained at 53% and 12% of their maximum value at 0.5 A/g current density for ASC-2, and ASC-3, respectively. The stability analysis further shows a capacitance retention of 89% and 64% at 0.02 A/g over 100 cycles for ASC-2 and ASC-3, respectively, which indicates a superior cyclic stability for the POC@Cu-ferrite-based ASC. Due to the monolithic nature of the electrode, packaging of ASC, and the drying issue associated with aqueous electrolyte, stability study beyond 100 cycles was not feasible [40]. At higher current densities, Faradaic reactions between the ASC electrode and electrolyte become sluggish, causing a reduction in specific capacitance [58]. The electrochemical stability of Cu-ferrite and POC@Cu-ferrite working electrodes at 0.5 A/g for 10,000 cycles is presented in Figure 9. The hybrid POC@Cu-ferrite electrode shows excellent stability with ~99% capacitance retention; however, the capacitance decreases by ~14% for Cu-ferrite.

**Figure 10:**
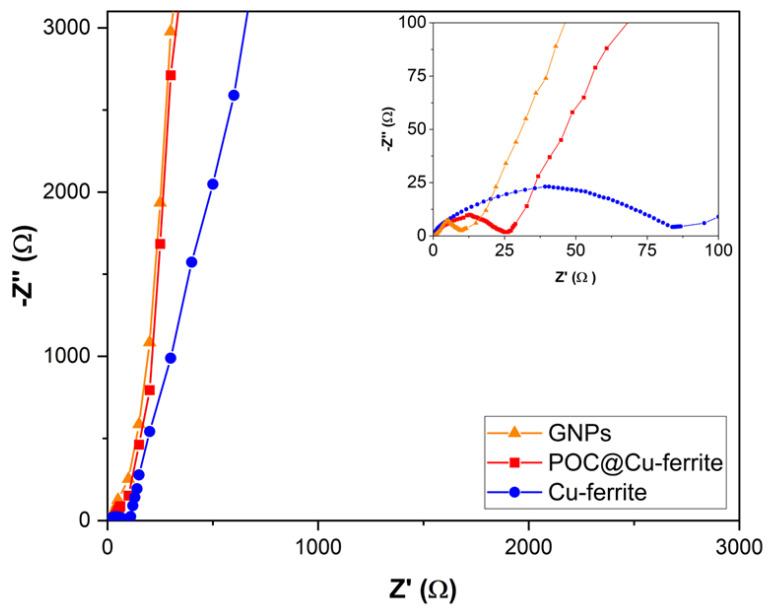
Nyquist plots for GNPs, POC@Cu-ferrite, and Cu-ferrite.

Electrochemical impedance spectroscopy (EIS) is employed to understand the resistance of electrode materials, and the interaction between electrodes and electrolytes. Nyquist plots within the frequency range of 0.01 to 30,000 Hz for graphene nanoplatelets (GNPs), POC@Cu-ferrite, and Cu-ferrite materials are shown in Figure 10, where Z′ is the real component and Z″ is the imaginary component of the impedance. It can be observed that all Nyquist plots show semicircles in the high frequency region, Warburg resistance at the middle frequency region, and linear lines along the imaginary axis at the low frequency region which can be possibly assigned to the interfacial charge resistance of electrode materials, the effect of electrode porosity on ion-diffusion from electrolyte to electrode, and the capacitive behavior, respectively [59]. Equivalent series resistance (ESR) can be determined in the high frequency region based on the intersection between the semicircles and the *x*-axis, shown as inset in Figure 10 [30,60]. The ESR value of Cu-ferrite is 83.2 Ω, while lower ESR value of 26.3 and 11.2 Ω was recorded for the POC@Cu-ferrite and GNPs material, respectively. The lower ESR value in the POC@Cu-ferrite material can possibly be attributed to higher surface wettability and extent of graphitization [61,62,63]. As for the Warburg resistance at the middle frequency region, a slope of 1 indicates an ideal ion diffusion between the electrode and electrolyte. GNPs, POC@Cu-ferrite, and Cu-ferrite show a slope of 1.71, 1.98 and 3.96, respectively. It is speculated that the porosity of the POC@Cu-ferrite material facilitated the electrolyte ion diffusion. Lastly, the capacitive behavior of electrode materials can be determined in the low frequency region of the Nyquist plot, in which a higher slope in this region corresponds to a better capacitive behavior [64]. In this region, values of 4.65, 4.11 and 2.54 were obtained for GNPs, POC@Cu-ferrite, and Cu-ferrite, respectively. POC@Cu-ferrite possesses a superior ion diffusion and capacitive behavior as compared to the Cu-ferrite material, suggesting enhanced electrochemical performance.

Overall, ASC fabricated with POC@Cu-ferrite displayed a superior electrochemical performance as compared to the Cu-ferrite material, which is evident from the CV, GCD, Nyquist plots, and stability analysis. In comparison to GNPs, POC showed inferior electrochemical performance. This may be due to lower degree of graphitization in POC. The specific capacitance of POC@Cu-ferrite was observed as 178 F/g, which is relatively similar to the copper-based working electrodes reported in the literature, and that include 185.1 F/g for tween-modified CuFe_2_O_4_ [21], 163 F/g for CuFe_2_O_4_-rGO [65], and 189.2 F/g for CuFe_2_O_4_ nanoparticles [49]. Table 2 compares the performance of the hybrid POC@Cu-ferrite electrode with recently reported electrode materials.

## 4. Conclusions

Porous carbon (POC) was successfully prepared from the corn stover-derived hydrochar and combined with Cu and Fe precursors via the sol–gel chemistry approach to obtain the POC@Cu-ferrite hybrid working electrode material. The BET analysis of POC@Cu-ferrite showed significantly higher SSA and pore volume with an average pore size of 4.5 nm as compared to Cu-ferrite. Raman spectroscopy of POC@Cu-ferrite showed I_D_ to I_G_ intensity ratio of 1.09 and crystal planer size of 4.0 nm. SEM/EDX analysis of POC@Cu-ferrite showed spherical morphology with an average particle size of 74 nm, and distributions of Cu and Fe on the POC surface. TEM/SAED analysis of POC revealed mesopores amorphous carbon with lattice d-spacing of 0.37 nm. The hybrid POC@Cu-ferrite working electrode material displays a maximum specific capacitance of 178 F/g at a current density of 0.02 A/g, which is higher than the specific capacitance of 85 F/g observed for Cu-ferrite. POC@Cu-ferrite and Cu-ferrite-based ASC exhibited a capacity retention rate of 89% and 64%, respectively, over 100 charging–discharging cycles. The working hybrid POC@Cu-ferrite electrode showed an excellent cyclic stability of ~99% with capacitance retention at 0.5 A/g current density for 10,000 cycles, whereas ~86% of capacitance retention was observed for Cu-ferrite. The reason for the superior performance of POC@Cu-ferrite appears to be the higher porosity and mesoporous structure that might have facilitated electrolyte ion diffusion and enhanced the capacitive behavior.

## Figures and Tables

**Table 1:** ASC prototype identification with different electrode materials.

ASC IDs	Electrode Materials (Cathode//Anode)
ASC-1	Cu-ferrite//graphene nanoplatelets (GNPs)
ASC-2	POC@Cu-ferrite//POC
ASC-3	Cu-ferrite//POC

**Table 2:** Comparison of hybrid POC@Cu-ferrite electrode material with previously reported working electrodes.

Electrode Material	Specific Capacitance (F/g)	Capacitance Retention (%)	Cycles	References
POC@Cu-ferrite	178	99	10,000	this study
Tween-modified CuFe_2_O_4_	185.1	90	2000	[21]
CuFe_2_O_4_-rGO	163	97	10,000	[65]
Spherical CuFe_2_O_4_ nanoparticles	189.2	84	1000	[49]
Grape-based honeycomb-like POC	275	95.2	5000	[66]
Nitrogen, sulfur co-doped pollen-derived carbon/graphene composite	420	94	10,000	[67]
ZnCl_2_ regulated flax-based POC fibers	105	98.7	10,000	[68]
3D carbonized polyimide/cellulose composite	300	91.4	10,000	[69]
α-Fe_2_O_3_ nanoparticles anchored on nitrogen-doped wood carbons	603	85.5	10,000	[70]

## Data Availability

The data that support the findings of this study are available from the corresponding author upon reasonable request.
